# Sibship Size, Height and Cohort Selection: A Methodological Approach

**DOI:** 10.3390/ijerph182413369

**Published:** 2021-12-19

**Authors:** Ramon Ramon-Muñoz, Josep-Maria Ramon-Muñoz, Begoña Candela-Martínez

**Affiliations:** 1 Department of Economic History, Institutions, Politics and World Economy, Faculty of Economics and Business, University of Barcelona, Diagonal 690, 08034 Barcelona, Spain; 2 Department of Applied Economics, Faculty of Economics and Business, University of Murcia, Campus de Espinardo, 30100 Murcia, Spain; jmramon@um.es (J.-M.R.-M.); bcandela@um.es (B.C.-M.)

**Keywords:** resource dilution hypothesis, family composition, quantity-quality trade off, biological living standards

## Abstract

This article deals with the historical relationship between the number of siblings in a family or household and height, a proxy for biological living standards. Ideally, this relationship is better assessed when we have evidence on the exact number of siblings in a family from its constitution onwards. However, this generally requires applying family reconstitution techniques, which, unfortunately, is not always possible. In this latter case, scholars must generally settle for considering only particular benchmark years using population censuses, from which family and household structures are derived. These data are then linked to the height data for the young males of the family or household. Height data are generally obtained from military records. In this matching process, several decisions have to be taken, which, in turn, are determined by source availability and the number of available observations. Using data from late 19th-century Catalonia, we explore whether the methodology used in matching population censuses and military records as described above might affect the relationship between sibship size and biological living standards and, if so, to what extent. We conclude that, while contextual factors cannot be neglected, the methodological decisions made in the initial steps of research also play a role in assessing this relationship.

## 1. Introduction

The impact of sibship size on children and young people’s nutrition and health status is not a minor topic. A burgeoning historical literature has recently discussed such a relationship within the framework of the resource dilution hypothesis, using height as a proxy for physical welfare [1]. The resource dilution hypothesis (RDH), which predicts a negative relationship between the number of sons and daughters in a family and child outcomes, assumes finite parental resources that tend to dilute as the number of children increases [2,3]. It also assumes that no resources come from outside the parents while resources remain relatively constant. Finally, it considers that siblings compete for the available resources and that the youngest and later-born children in a family face more intense competition since resources tend to dilute as the number of sisters and brothers increases.

Parental resources are crucial in children’s development and physical growth [4], especially during the early years of life [5]. In addition to genetics, environmental and nutritional factors are also critical in the final height of adult populations (e.g., [6,7,8,9,10,11]). Thus, it might be hypothesized that greater competition between siblings for family resources can lead to food deprivation and, consequently, to shorter heights.

There is an ample number of historical studies that support the resource dilution hypothesis. In northern Europe, the negative effect of sibship size on height was found in both England and Wales for men born in the 1890s [12,13] and for children born in the 1920s and 1930s [13,14,15]. This negative relationship was also found in Sweden among recruits born between 1881 and 1921 [16] and in the Dutch province of Drenthe for young people born in the first half of the 19th century [17]. By exploring the relationship between family size and birth order to height, similar conclusions were obtained for the cohorts of Dutch conscripts born between 1944 and 1947 [18], and for Swiss recruits born between 1951 and 1983 [19]. In southern Europe, a number of studies also confirm this negative relationship between the number of siblings in a family and the physical stature of their drafted members [20,21]. Finally, in the same vein, in southeastern Minnesota, sibship size was found to have had a strong influence on the height of children born throughout the first two decades of the 20th century [22].

However, it is far from universal that a larger number of siblings—and other indicators of sibship and family size—lead to a lower biological living standard for the children living in a family or household. An example of this is provided by Beekink and Kok, who found that family composition did not significantly affect the physical stature of Dutch recruits from the province of Utrecht born in the early 19th century [23]. Similar conclusions were obtained for the cohorts of young males born between the mid-19th and early 20th centuries in central-eastern Sardinia [24] and in central Catalonia [25]. In addition, studies that consider the birth order of siblings and have correlated it with height data have found no relationship between these two variables, as is the case of English and Welsh men conscripted into the army in the First World War [12] and Dutch conscripts born between 1944 and 1947 [26]. Finally, it has also been shown that the negative relationship between sibship size and stature tends to weaken over time and varies by social groups and gender and depending on the environmental context [16,17,27,28].

In short, at the present research stage, it seems evident that when stature is related to family size, the results we obtain do not always support the RDH [1]. The relationship between sibship size and height is more context- and time-specific than was probably expected initially because it is influenced by a series of confounding factors [25]. To some extent, one might conclude that a more flexible approach is more suitable when testing the dilution hypothesis, as the conditional resource dilution hypothesis suggests [29].

While the relationship between sibship size and height may vary over time and across space, the conclusions reached by scholars who have addressed this topic have been obtained using different approaches and methodologies. Ideally, this relationship is better assessed when we have evidence on the exact number of siblings in a family from its constitution onwards. However, this generally requires applying family reconstitution techniques. It is well known that this method involves the longitudinal construction of individual life histories using records of demographic life-course events (such as baptisms, burials and marriages) [30,31]. Unfortunately, it is not always possible to implement this technique. In this latter case, scholars must generally settle for considering only particular benchmark years, mainly using population censuses, from which family and household structures are derived. This information is then linked to the height data for the young males of the family. In this matching process, several decisions have to be made, which, in turn, are determined by source availability and the number of available observations.

Being a second-best option, this latter method of connecting data may cause a loss of information. While it allows us to identify the number of siblings living in the household in the census year, it does not provide any information about any siblings that may have died in the early years of life and prior to the census year to which anthropometric data are linked. Thus, it is possible that, with this technique, the effect of resource competition between siblings is underestimated, which can be especially problematic for contexts or socioeconomic groups with higher mortality rates or greater population mobility through migratory processes [25].

This article discusses the potential impact of certain methodological issues when testing for the historical relationship between the number of siblings in a family or household and their biological living standard. In particular, the main aim of this paper is precisely to assess some of the potential shortcomings that, in the absence of family reconstitution, might emerge by using this alternative methodology that derives the structure of the recruits’ families for a particular point in time. As other scholars might use this methodology in the future, we believe that the discussion of this methodological issue, which has not yet been addressed, might provide valuable insights on the topic [1]. This is particularly true when scholars have to deal with limited family information due to data scarcity. We consider a medium-sized town in central Catalonia as a case study. We use family and household data obtained from the 1890 local population census and link these data to the military information collected for the cohorts of young men born between 1871 and 1890. We, therefore, discuss an extreme case, namely when scholars can only make use of family information for a particular single point in time.

The rest of this article is organized as follows: Section 2 reviews and discusses the historical literature linking height data with family information by focusing on particular methodological aspects; Section 3 focuses on contextualizing our case study and describing the primary sources and data that we will be using in this research, as well as some preliminary results that pave the way for the following sections; Section 4 presents the econometric model we apply; Section 5 discusses the results we have obtained from our econometric exercises; and finally, Section 6 draws some conclusions, which should be interpreted with some caution.

## 2. Literature Review

For the analysis of the historical impact of family and household size and structure on individuals’ height, scholars have used several sources. Regarding height data, most studies have obtained the physical stature of individuals from military records, although information from prisons, schools and hospitals has also been used. Alongside the physical stature of the draftee, his year of birth and place of residence, military sources may provide additional pieces of evidence, which may include, among others, the place of birth, profession and literacy level of the draftee, as well as his parents’ names and other parental information. In contrast, military records do not specify the recruits’ family and household size and structure. Thus, this information must be obtained from alternative demographic sources, generally consisting of civil and church records of births, marriages and deaths, and nominal population censuses. With this demographic information, family and household structures are derived and, finally, linked to the physical stature of the focal individual using nominative linkage techniques.

In general, scholars have followed two different approaches in linking height data with demographic information (see Table 1). The first approach is based on family reconstitution techniques and, consequently, considers all the demographic events that occurred in a family from the moment it was formed onwards. The second approach only takes into account the structure and size of the family at a particular time point.

The first approach is ideal for investigating the association between sibship size and biological living standards [4,32]. The height that individuals reach in adulthood is strongly determined in early infancy and even in the intrauterine stage, but it is also sensitive to circumstances during childhood, adolescence and early youth. Once the family is formed, family reconstitution allows us to identify the composition and characteristics of families from the time one of their members is born until he or she leaves the household. In other words, if we have to assess whether or not family size influences the height of an individual, it is better to have access to longitudinal information that captures the size and other characteristics of the family at every moment in time for which the focal individual belongs to the family.

Technological advances have permitted the construction of large-scale longitudinal databases based on family reconstitution [33,34]. The Scanian Economic Demographic Database (hereafter SEDD) and the Historical Sample of the Netherlands (hereafter HSN) are two clear examples in northern Europe. In both cases, these are longitudinal datasets derived from active registration that, in addition, avoid the sub-register problems that migratory movements can generate. Furthermore, family members are followed and monitored over time, with different historical events being recorded as they occur, and, therefore, continuous information is provided [31]. The potential of the SEDD [35] and the HSN [36,37,38] datasets for studies aiming at assessing the association between family size and biological living standards is apparent compared to alternative methods and, for this reason, they have already been used for this purpose. Two excellent examples of using these datasets are the studies by S. Öberg [16] and B. Quanjer and J. Kok [27]. The former focuses on five rural parishes in southern Sweden. The latter analyzes the eight provinces of the Netherlands (i.e., Friesland, Drenthe, Overijssel, Noord-Holland, Zuid-Holland, Utrecht, Brabant and Limburg).

Although not all countries have developed datasets like the SEDD and the HSN, this has not been an obstacle for scholars to construct local datasets using family reconstitution techniques. For example, for the Mediterranean island of Sardinia, S. Mazzoni, M. Breschi, M. Manfredini, L. Pozzi & G. Ruiu [20] focused on the case of L’Alguer (Alguero), a coastal town located in northwestern Sardinia; and M. Poulain, D. Chambre, A. Herm & G. Pes [24] analyzed the case of Villagrande Strisaili, a town in the central-eastern part of the island. In both cases, these scholars carried out longitudinal studies based on family reconstitution. The same applies to the research by E. Beekink and J. Kok [23], and by J. Kok, E. Beekink and D. Bijsterbosch [28], this time for the Netherlands. Beekink and Kok considered the case of Woerden, an industrial town located in the province of Utrecht. In Kok, Beekink and Bijsterbosch, this industrial town was compared to Akersloot, a rural, agrarian village in North Holland. For Akersloot, the authors used demographic data from the HSN.

While the studies considered so far were able to take advantage of existing or newly constructed datasets based on family reconstitution techniques, another group of works has followed a different approach. In particular, they have considered the structure and size of the family at a particular point in time, which is then connected to military information. Primarily based on the use of population censuses, this second approach should be considered a second-best option in the absence of datasets or the required sources to reconstruct family life courses.

Several studies fall into this second group. They include, for example, the works of R.E. Bailey, T.J. Hatton & K. Inwood [12] (see also [13]) for England and Wales, E. Roberts & J.R. Warren [22] for the American city of Saint Paul (Minnesota), R. Ramon-Muñoz & J.-M. Ramon-Muñoz [25] for the case of Igualada, an industrial town in central Catalonia and G. Galofré-Vilà [21] for the northeastern Catalan city of Girona. However, although all these authors followed a similar general approach, there are also some methodological differences among them when linking individual anthropometric data with household and family data (see Table 1).

The use of population censuses is not universal among scholars who considerer only particular benchmark years. T.J. Hatton and R.M. Martin [14] (see also [13]) for England and Scotland and L. Stradford, F. Van Poppel & L.H. Lumey [26] for the Netherlands provide examples in the use of alternative data sources. In these two cases, the required information was obtained from a single source providing both height data and information on family composition. In contrast, by analyzing two communities in the Dutch province of Drenthe in the first half of the 19th century, V. Tassenaar and E.E. Karel’s analysis [17] followed a different strategy for data collection. They used military records for obtaining height data, but derived family information from different sources.

To sum up, in the absence of datasets and other limitations to making use of information based on family reconstitutions, researchers have generally used alternative methods and sources in the process of matching anthropometric data with demographic information (Table 1). On most occasions, this method has involved no more than linking individuals, generally young people and children, of a certain group of cohorts to a specific population census or a specific point in time. In these cases, the dynamic composition of families and household cannot be considered. Thus, some scholars have reconstructed or used family and household structures at the age at which the individual was medically inspected. Other scholars have reconstructed the characteristics of individuals in the household when they were around 10 years old, although the actual individual’s stature might correspond to a different individual’s age (Table 1). For example, in this case, they might be referring to a young man born in 1880, measured in 1900, when he was 20 years old, but linked to the population census in 1890, when he was 10 years old.

Useful as they can be, these methods of matching raise two central and closely interlinked questions, particularly when military sources are used. The first relates to the age of the recruit that we should take as a reference to establish the size, composition and characteristics of the family and household under consideration. There is not a general pattern, as shown in Table 1. While some studies prioritized the moment at which the recruit was around the age of 10, there are no clear reasons to consider that other ages might not be equally suitable for establishing the impact of sibship size on biological living standards.

For example, the early childhood years might be another potential possibility. Indeed, the growth velocity of height is higher in the first years of life than later on [39]. Thus, it might be argued that, in addition to environmental conditions, the household characteristics in which individuals live during the early years of their life will strongly determine their final adult height, which most anthropometric studies assume. Puberty, and the conditions in which this occurs, is another period in which physical stature accelerates. These are the years when the pubertal growth spurt takes place. Therefore, it might also be argued that family conditions during this period may also influence final adult stature. To add complexity to the pubertal growth spurt, in historical populations, this occurred later than nowadays. In this respect, P. Gao and E.B. Schneider [40] have observed that, in the cohorts born in Britain prior to the First World War, the pubertal growth spurt was not as evident as may be thought. As these authors pointed out, “growth velocity between ages 12 and 17 was relatively low at between four and five centimeters per year, and there was no marked pubertal growth spurt as the growth velocity was similar across these ages” [40] (p. 356). In contrast, for the decades after 1910, the stature’s growth velocity accelerated relative to previous periods while it also took place in a short period, between the ages of 14 and 16 years. Of course, these findings do not mean that the physical stature of young people was not growing after the age of 16 years; and there is ample evidence showing that, in the 19th century, young males reached their final height at the ages of around 22–23 years (e.g., [28,41,42,43]). They instead contribute to showing the historical dimension of the pubertal growth spurt.

The second question arising from the matching process is strongly connected to these previous comments. In the absence of longitudinal information based on family reconstitution, one might wonder whether the relationship between sibship size and stature is sensitive to the year of birth of the selected cohort. Consider as an example that we can only use a single population census, such as the one conducted in 1890. Moreover, consider that the height information at our disposal refers to a certain number of cohorts, e.g., the cohorts born in 1886–1890 (individuals at the age of 0 to 4 relative to the 1890 population census), 1881–1885 (age 5 to 9), 1876–1880 (age 10 to 14) and 1871–1875 (age 15 to 19). Finally, consider that we have to select one of the groups of cohorts in order to test for the relationship between sibship size and physical stature. Will the results vary depending on the groups of cohorts we select? This is precisely the question we aim to answer over the course of this article. It may undoubtedly be seen as a relatively simple question, although the answer to this question might have some relevant implications.

## 3. The Case Study: Context, Sources, Data and Preliminary Results

**Figure 1:**
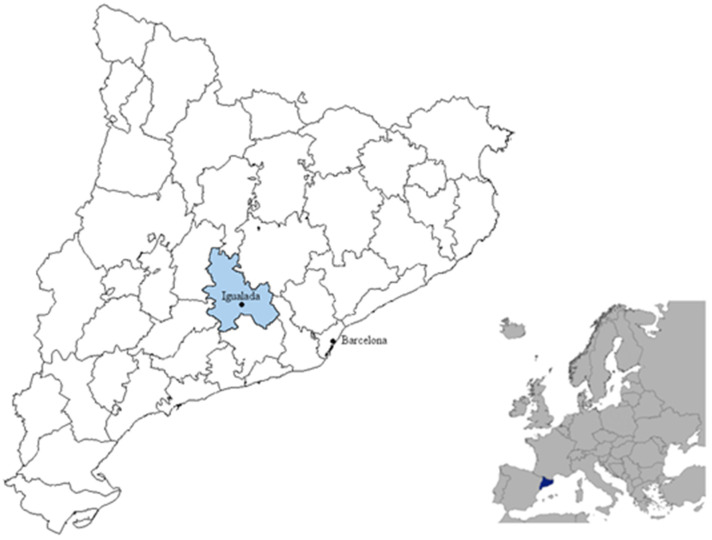
The geographical location of Igualada. Sources: based on http://epp.eurostat.ec.europa.eu and http://municat.gencat.cat (accessed on 19 July 2016).

Our research strategy consists of answering this question through a case study. Therefore, this section is devoted to presenting the case and providing general information on our primary sources and data. We focus on the Catalan town of Igualada in the late 19th century. There are several reasons for our choice. Firstly, Igualada was a medium-sized manufacturing town located at the center of a leading industrial region in southern Europe (Figure 1). To a certain extent, it can be considered representative of the urban and industrial society emerging in 19th-century Catalonia. It also illustrates the cotton- and factory-based industrialization process rooted in wool protoindustrial manufacture. After rapid economic and demographic growth, mainly driven by immigration, the town suffered a severe crisis around the mid-19th century [44,45,46,47,48]. The population dropped from 14,000 to around 10,200 inhabitants between the census years of 1857 and 1887. However, the town never lost its industrial orientation. More than half of the adult population was still employed in the secondary sector in the latter census year. The same was true in 1900 when the number of inhabitants was almost 10,450 and economic recovery was already under way.

**Figure 2:**
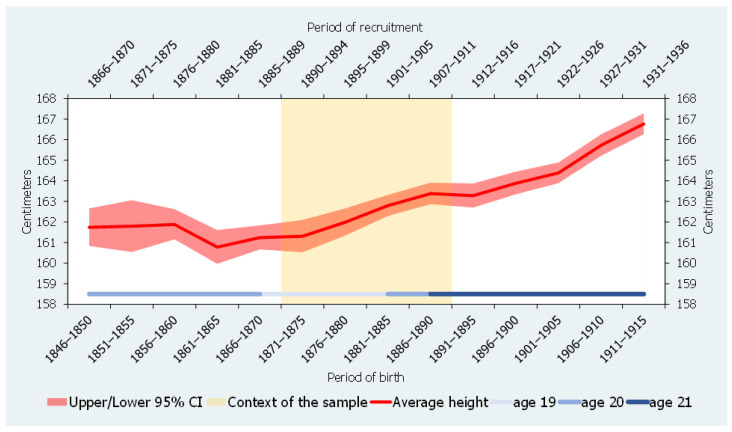
Average height of conscripts in Igualada, 1846–1915 (five-year annual averages, in centimeters). Source: Ramon-Muñoz and Ramon-Muñoz [25,48].

Secondly, the case of Igualada is also interesting as, over the last decades of the 19th century, the town was experiencing the second phase of the demographical transition. Between the early 1860s and the early 1890s, the number of baptisms by marriage declined from more than 5 to 4 [25] (p. 338). As a result, the average family size also dropped. This drop may indicate that industrialization and, notably, the massive arrival of immigrants at the beginning of the 19th century might have fostered the development of a nuclear model of family organization. Moreover, the increase in the number of nuclear families may have been reinforced by a long-term decline in the marriage age that took place in Igualada, especially for men [49]. The relationship between nuclear families and immigrant families has been observed in other Catalan industrial areas. However, over the years, these nuclear families became stem families, which was, in fact, the predominant system in Catalonia through the figure of the “*hereu*” (heir) [50,51,52,53]. Interestingly, the reduction in birth rates and family size ran parallel to an increase in the average height of young males, which rose around 2.5 cm over the same period, from 160.8 to 163.3 cm (see Figure 2). In addition, population density also declined, and, thus, it might be hypothesized that the observed improvement in biological living standards was connected to changes in family structure.

Thirdly, previous studies have already focused on biological standards of living and sibship size in this town and for a similar period. Therefore, the background of this particular case study is well established. Our research takes advantage of (and departs from) these former studies [25,48], thanks to which, we know that military sources with height information are available for Igualada for this period, and also that it is feasible to link them with the local population censuses preserved from the end of the 19th century. In particular, the data on heights are provided by the Actas de clasificación y declaración de soldados (Acts of classification and declaration of soldiers) for the period 1890–1911 (Arxiu Comarcal de l’Anoia, Archive of the county of Anoia, hereinafter ACAN). The demographic and socioeconomic data are provided by the local population census of 1890, known as the Padrón general de vecinos (Ayuntamiento constitucional de la ciudad de Igualada, Igualada, Imprenta y Fábrica de Rayados de Mariano Abadal, ACAN). These data form the basis of our analysis and, as usual, the former have been linked to the latter. Table 2 reports the number of recruits registered and the percentage of conscripts with height data found in the 1890 population census. Starting from an initial dataset of 1918 recruits, we had to discard 265 as height data were not reported in the source. Of the remaining 1653 recruits, we were able to link 70 per cent with their families successfully, a matching ratio in line with that obtained in other studies (e.g., [16,20,22,25]). In total, our dataset comprises 1157 young males and 774 families.

**Figure 3:**
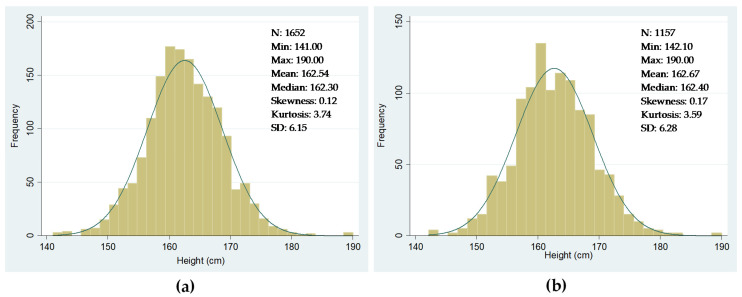
Distribution of heights of conscripts from Igualada (in centimeters). (**a**) All conscripts’ distribution of heights (cm); (**b**) full sample distribution of heights. Sources: see text in this section.

Potential shortcomings in our dataset cannot be excluded. Nevertheless, if they exist, they do not appear to be significant enough to bias our results. As far as the military sources are concerned, in the period we are considering, military service was universal and compulsory in Spain, although the existing legislation still allowed the substitution of recruits through cash payments and redemptions [54]. However, the recruit’s substitution took place after the inspection process had ended, which means that the local authorities collected all heights during measurement and, therefore, our data are not affected by this potential issue. Figure 3 shows the frequency distributions of heights. Figure 3a considers all the conscripts born between 1871 and 1890 as reported in the military sources. Note that the total number of observations with height data is now 1652 rather than 1653, (see Table 2), as we excluded a 102 cm tall recruit. Figure 3b only considers the conscripts that we were able to link to their families using the 1890 population census. As observed in both figures, the distribution of heights is quasi-normal, confirming that our data were not affected by truncation or probably by any other severe potential bias. The data presented in Figure 3 also include the heights of recruits measured at different ages due to changes in the recruitments’ enlistment age. Despite this, we decided not to standardize height data but to control for changes in the age of enlistment when performing econometric exercises.

Regarding demographic information, we decided to choose the 1890 local population census as it is the first of a regular collection of censuses for the late 19th century that allows the construction of family structures with an acceptable, though imperfect, degree of accuracy. However, this census is not free from problems, as is probably the case for all those available for late 19th-century Igualada. The most critical problem we faced was the under-registration of the female population, i.e., the absence in the family of the recruit’s mother and any potential sisters. For example, our dataset reports that girls accounted for around 30 per cent of the total number of children and young people (recruits and their siblings) present in the households in the census year of 1890. In order to mitigate these shortcomings, we had to accept a partial solution, which could only be applied to the recruits’ mothers. Thus, when the recruit’s mother was not reported in the population census, we looked at the marital information of the recruit’s father, and we assumed that if the father was registered as a married male, the mother was also present in the household, irrespective of whether the census reported her presence or not. Unfortunately, no correction was possible regarding the likely absence of the potential sisters of the recruits.

The matter of the missing girls is complex due to the lack of in-depth research referring to Igualada. At the present research stage, we can only suggest that sub-registration is just one of the potential explanations of the low ratio of girls. In this respect, discriminatory practices have been documented for 19th-century Spain, leading to excessive female mortality in early life [55,56]. According to F.J. Beltrán and D. Gallego, excessive female mortality was probably related to an unequal distribution of resources within the household, with girls suffering from gender discrimination. For these authors, only the demand for female wage labor and the prevalence of stem families had the power to reduce gender discriminatory practices. Female labor demand tended to be high in the local textile industry, the leading industry in Igualada in the 19th and early 20th centuries [57,58,59]. However, this sector also experienced periods of crisis; therefore, it might be hypothesized that, apart from or as well as gender discrimination, the issue of the missing girls may be connected to the fact that they had left their families to work as servants elsewhere, at least in specific periods. In the following sections, we shall return to the matter of the missing girls and its effect on our results. For the time being, it is sufficient to say that it is doubtful that our results were significantly biased by this issue.

**Figure 4:**
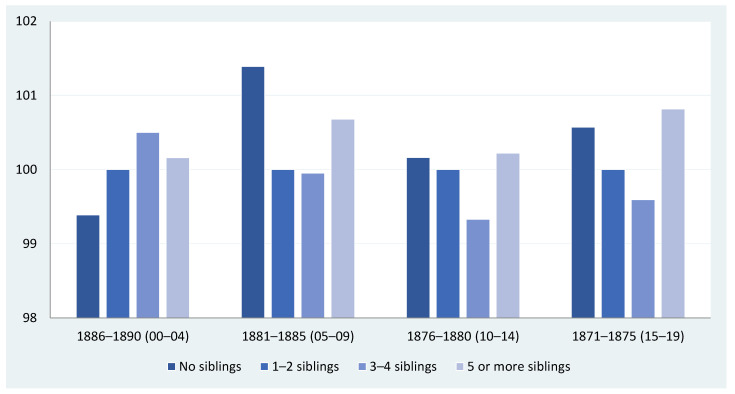
Sibship size and height in Igualada (Catalonia) by cohort groups of five birth years (conscripts with 1–2 siblings = 100). Sources: see text in Section 4.

**Figure 5:**
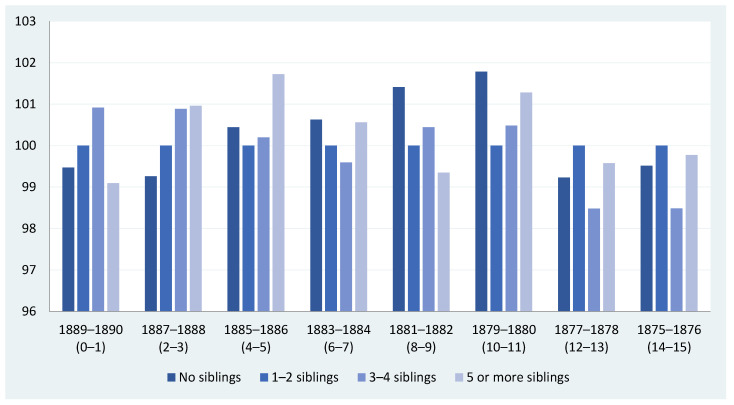
Sibship size and height in Igualada (Catalonia) by groups of two cohorts (conscripts with no siblings = 100). Sources: see text in Section 4.

So far, we have presented the context, the sources and the data we will be using. What do they tell us about the association between the number of siblings and the biological living standards in late 19th-century Igualada? Does this association vary depending on the groups of cohorts we select when linking them to a particular population census? Figure 4 and Figure 5 provide some preliminary answers to these questions and pave the way for further analysis. We have organized our military data in four different groups of cohorts, and we have linked them to the focal recruit’s family to ascertain the number of siblings the recruit had according to the 1890 population census. The family information is time-invariant, while there are four groups of cohorts ranging from those closer to the 1890 population census (birth cohort of 1886–1890), when the recruits were between 0 and 4 years old in 1890, to those farther away from our reference census, when the recruits were between 15 and 19 years old in 1890. In Figure 4 and Figure 5, the conscripts’ age is in brackets. Finally, the data shown in these figures are expressed in index numbers to make comparisons between different birth cohorts easier, with the average height of the group of recruits with one and two siblings being equal to 100.

Three main points emerge from the crude evidence presented in Figure 4. Firstly, there appears to be a weak linear relationship between sibship size and physical stature. In most cohort groups, this weak linearity is mainly to do with the fact that the height of conscripts with five or more siblings breaks down the linear relationships we generally observe for other sibling categories.

Secondly, the RDH predicts a negative association between sibship size and height. In our case study, however, this relationship is unclear and far from universal. However, the conditional resource-dilution model provides a conceptual framework that might help us to understand why the absence of clear negative trends is feasible under certain circumstances [29,60], and we shall return to this issue later in this paper.

Thirdly, there is no clear pattern in the relationship between sibship size and height; therefore, heterogeneity prevails. For some of the birth cohorts closer to the 1890 population census year, we observe that the number of siblings positively impacted height. In the birth cohorts of 1886–1890, physical stature increases with the number of siblings, although, after the fourth sibling, it declines. For the birth cohorts of 1881–1885, conscripts with no siblings are taller than those with one to four siblings, but the height of conscripts with more than four siblings increases. In contrast, for the groups of cohorts born between 1871 and 1880, aged between 10 and 19 years at the time of the 1890 population census, the relationship is negative, at least up to the fifth sibling. After that, the mean height of the conscripts with five or more siblings is higher and, in fact, surpasses the stature of those who do not have siblings.

Figure 5 shows the same information as Figure 4. However, instead of dividing our sample into groups of five consecutive birth cohorts, we split it into groups of two consecutive birth cohorts. Perhaps not surprisingly, the heterogeneity in the relationship between sibship size and physical stature is now more apparent, but we arrive at similar conclusions as in Figure 4.

Taking this preliminary evidence as a whole, we might provisionally conclude that the relationship between sibship size and height appears to be sensitive to the group of birth cohorts we consider. By using econometric techniques, the remainder of this study will try to assess whether this hypothesis can be confirmed or not and, if so, to what extent.

## 4. Econometric Model

To disentangle whether the relationship between the number of siblings and the physical stature of individuals is sensitive to the cohorts we select, we use a basic econometric model and run OLS multiple linear regressions. The primary dependent variable of our model is the conscripts’ height. The independent variable of main interest is the number of siblings the conscript has. Additional control independent variables include information on the composition and characteristics of the conscripts’ household, including parental presence in the household, the conscripts’ place of birth and their father’s literacy and occupation. In Table 3, we present a summary of the main descriptive statistics of our sample, which consists of a total of 1157 observations.

**Figure 6:**
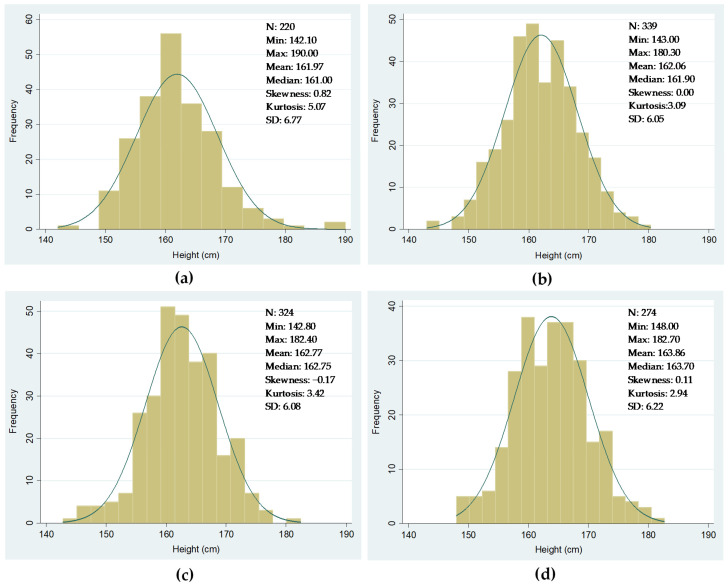
Distribution of heights of conscripts from Igualada by cohort group (in centimeters). (**a**) Cohort group born 1871–1875; (**b**) cohort group born 1876–1880; (**c**) cohort group born 1881–1885; (**d**) cohort group born 1886–1890. Sources: see text in this section.

In our exercises, we keep the original family structures constant, according to the 1890 population census. Instead, we cluster the conscripts in four different groups of birth cohorts, including recruits aged from 0 to 14 years at the time of the 1890 population census. From this census, we obtain information on the number of siblings and other data about the family and household. Therefore, we assume that the number of siblings is a time-invariant variable referring to a particular moment in time. Figure 6 shows the frequency distribution of heights corresponding to these four five-year birth cohort groups. These distributions follow a quasi-normal pattern, as is the case for the whole sample of this study (Figure 6).

*Height*_*jt*_ = *β*0 + *β*1*Sibsize_jt_* + *βZ_jt_* + *μ_jt_* (1)

Equation (1) summarizes the basic econometric model we use for estimation analysis. *Height_jt_* is the dependent variable and contains information about height in centimeters of the recruit *j* in year *t*. The main explanatory variable in the equation is the number of siblings of conscript *j* in year *t* (*Sibsize_jt_*). In addition, we include a series of control variables denoted as *Z_jt_* in Equation (1).

The main explanatory variable is entered into the regressions in two different forms: firstly as a continuous variable and secondly as a categorical or, more precisely, dichotomous variable (Table 3). In this latter case, we have dummies depending on the number of siblings. For example, 1 if the recruit has no siblings, 0 otherwise; 1 if the recruit has 1 or 2 siblings, 0 otherwise, and so on. The inclusion of these dummy variables responds to a potential weak linear relationship between sibship size and height, as other studies have previously shown [61,62], and our preliminary results suggest (Figure 4 and Figure 5). However, we are also aware that approaching sibship size through the number of siblings might be problematic; it might cause simultaneity and endogeneity biases. This is because of the likely correlation between parents’ decisions about how many children to have and how much to invest in each child. Empirical strategies based on the use of instrumental variables have been used to try to solve this issue, and scholars have generally used twins’ births as the instrument for sibship size [63,64,65]. However, our research cannot implement this instrumental variable strategy as there are hardly any twin siblings in our dataset. Furthermore, some researchers have expressed doubts about the validity of this instrument [26,66,67]. In any case, our main research aim is not to identify causal effects. Instead, we mainly focus on the extent to which differences in the model specification throughout the cohort selection can result in differences in the regression results.

**Figure 7:**
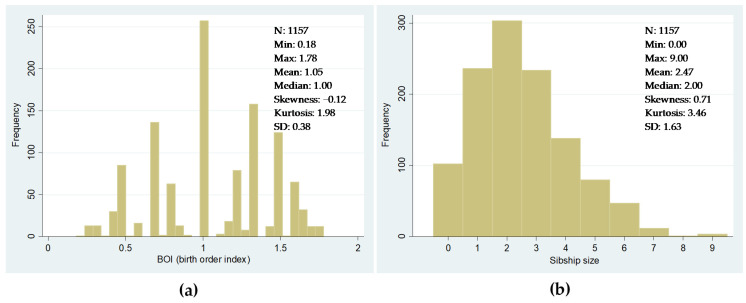
Distribution of the birth order index (BOI) and the sibship size in the dataset. (**a**) BOI; (**b**) sibship size. Sources: see text in this section.

As far as the rest of the independent variables are concerned, we included the following control variables in all model specifications. Firstly, we included a birth order index to capture specific characteristics of each child. In the context of the resource dilution hypothesis, this index can explain differences in access to family resources. We calculated this index based on the birth order index created by A.L. Booth and H.J. Kee [68], which avoids possible collinearity problems arising from the dependent relationship between sibship size and order of birth. This index, which has also been applied in other historical studies [16,25,27], is constructed from the expression: (BOI) = Birth order/((number of Children + 1)/2). Figure 7 shows the frequency distributions of the BOI and the sibship size distribution in our dataset.

Apart from the BOI, we included in our model a dummy variable that aims to capture whether or not both parents were alive in the census year of 1890. We considered it essential to control for the parental circumstances due to the importance of parental resources in relation to child development [69,70]. These resources involve material goods, such as providing food for their growth and development, and non-material resources, e.g., the time they spend with their children. Furthermore, we should expect a positive relationship between the presence of both parents in the household and children’s height, as we consider that height is an indicator of net nutritional status and biological living standards [71,72]. In contrast, we decided not to include a dummy variable that only captures the mother’s presence in a family, as has been done in other studies [27]. This decision is a consequence of the under-registration of females in the population census used and our approach to dealing with this issue (see Section 3). In this regard, we did not include any dummy variable to control the potential impact of the low presence of girls in the households on the relationship between sibship size and height. However, we carried out separate regressions using only information for male siblings, and the results were very similar to the ones we obtained when we used all the siblings, both boys and girls, present in the family.

Similarly, we did not consider whether or not a household had additional family members, such as grandparents and other relatives. Our dataset has 159 households with one or more additional members alongside the parents and their offspring. Although this is a relatively low number, we decided to check for its potential impact on our results. Thus, we firstly ran regressions of our baseline model by including a variable controlling for additional household members. The regression results showed that this variable was not significant for any of the birth cohorts we considered in our study. This lack of significance may be the consequence of the heterogeneous composition of the additional household membership, with members who contributed to the resources available in the household and individuals who were not productive and, therefore, competed in the family resources’ distribution. To capture this diversity, we then constructed a variable that consisted of the ratio between productive and consumer members within the household. Our results suggest that this ratio was neither coherent nor significant, contrary to what other researchers have found [27]. We certainly believe that we were unable to construct a solid ratio as we do not always have information about the occupation of all the household members. Therefore, we finally decided not to include variables related to the presence of additional members in a household in our regressions.

Instead, we considered it more relevant to capture potential differences in the literacy level and socioeconomic circumstances. The population census gives information about these factors, which we included in the regressions through dummy variables. Thus, we included a dummy that takes value 1 if the father knows how to read and write and 0 otherwise. As previous studies have shown, the parents having a higher educational level can result in better healthcare and nutrition for their offspring [73].

Concerning the recruit’s father’s occupation, we included this information in the regressions because, among other factors, it can solve the endogeneity problem that arises due to the existence of non-observable determinants in the parents’ preferences. To add this data in the regressions, we created dummy variables based on the historical grouping made by the HISCLASS classification [74]. These variables allow us to classify the working population into non-manual workers, manual workers with high-medium skills and manual workers with low skills or unskilled. In our sample, we have 152 observations for which we do not know the fathers’ occupation. These occupational variables can also be a potential source of collinearity, so we decided to use the dummy for fathers with non-manual occupation as the reference category. The results would be very similar if we selected other reference categories. Previous research found that differences in the parents’ socioeconomic characteristics, as proxied by occupation, could be critical and even more relevant than sibship size in explaining height differences across individuals [20,23].

The last two control variables in our empirical specification refer to the focal recruit’s place and year of birth. Thus, we included a dummy to control for conscripts who were born in Igualada or elsewhere. We also included birth year dummies that control for the different ages at which conscripts were measured.

Finally, we clustered the standard errors at the family level to adjust the expected correlations between siblings in running the regressions. Thus, the results of the estimations are robust to the heteroscedasticity of the errors. We also checked the normality of the residuals, and there is no evidence to reject the hypothesis that the errors show a normal distribution.

Table 4 shows the mean and standard deviation for height and sibship size by control variables. Descriptive statistics show that conscripts who had both parents alive were on average taller than the rest and had a slightly higher number of siblings. The same can be said for conscripts whose father was literate compared to those with non-literate fathers. Interestingly, the offspring of non-manual workers were around 2–3 cm taller than those of manual workers and had a slightly larger sibship size. With respect to the place of birth, differences in the mean height and the number of siblings are minimal between those born in Igualada and those born elsewhere. Finally, Table 4 captures the intergenerational increase in average heights that paralleled the reduction in the average number of siblings, although changes in the recruitment age also influence this upward trend in height.

## 5. Main Findings: Description and Discussion

This section describes and discusses the results obtained in the econometric model we presented in the previous section. These results are reported in Table 5 and Table 6, which only provide information for our primary variable of interest, namely the number of siblings of the considered recruits. However, we also included control variables in the regressions we ran (see Section 4). Furthermore, we also estimated two different specifications for sibship size; in the first, this variable is entered in the regressions as a continuous variable (panel 1) and, in the second specification, it is entered as a categorical variable (panel 2).

Table 5 and Table 6 confirm our preliminary findings, at least in part. The econometrical exercises point, in general, towards a negative relationship between sibship size and the biological living standard, as the dilution resources hypothesis would predict. However, this negative relationship is weak in the case of Igualada, and it is never statistically significant. Moreover, the results of the econometric exercises show that the association between the number of siblings and the physical stature of the recruits is not always characterized by a negative sign; in fact, a positive association is found for most of the cohorts of recruits born closer to the 1890 population census, although again, the association is not statistically significant.

In addition, we do not always observe homogeneous patterns for the cohorts in which our variable of interest has a negative sign. For example, recruits with no siblings tend to be taller than their counterparts, even though this difference is only statistically significant for the birth cohort of 1881–1885. Probably against expectations, recruits with more than five siblings are also generally taller than those with three and four siblings, although some exceptions emerge among the groups composed of two-year birth cohorts. Nevertheless, statistical significance is never found. Recruits with three and four siblings tend, in turn, to be shorter than those with one and two siblings, with the differences only being statistically significant for the cohorts born in the second half of the 1870s when the focal recruit was between 10 and 15 years old, but not for the rest of the cohorts. Regarding these latter cohorts, a different and probably unexpected pattern emerges for the conscripts born in the early 1880s: young males with three and four siblings are taller than those with one and two siblings.

The magnitude of the coefficients obtained in the regressions also shows remarkable heterogeneity, while no clear patterns for this heterogeneity emerge. This conclusion applies when our primary interest variable enters the regressions as a continuous variable (panel 1) or as a categorical variable (panel 2).

To sum up, in linking different birth cohorts of recruits to a time-invariant population census, the relationship between sibship size and height substantially varies across cohorts. However, it is also true that the results of the econometrical exercises show a clear common pattern for all our cohorts: the primary variable of interest, namely the number of siblings, generally lacked statistical significance. Nevertheless, despite this common pattern, diversity and variety still prevail.

Therefore, the question seems obvious: what explains cross-cohort heterogeneity in the relationship between the number of siblings a recruit has and his biological standard of living? We are well aware that several factors can explain the heterogeneity observed in our results. Though similar among them, the cohort size might be a potential source of variability. However, to minimize this potential impact, we have consistently established a threshold of 100 observations in all the regressions we have run. Moreover, we have always used samples of more than 200 observations when considering groups composed of five consecutive birth cohorts.

The second source of heterogeneity is related to the year in which conscripts were inspected and measured. Our dataset consists of recruits measured at 19, 20 and 21 years of age, respectively (see Table 7). This difference in the age of inspection might not be a minor issue. In the 19th and early 20th centuries, the stature of young people could continue increasing considerably after the age of 19, particularly among recruits that had experienced nutritional deficiencies or other adverse circumstances during childhood or adolescence. For eastern Belgium, G. Alter, M. Neven and M. Oris [75] suggested that, between the age of 19 and adulthood, physical stature could increase by around 3 cm. For 19th-century southern Europe, the rise in height might follow a different pattern. For example, in Catalonia, J.M. Ramon-Muñoz [76] found a height difference of 1 cm when the conscripts measured at 19 were compared to those inspected at 21 years old. Our study shows a slightly higher increase in height (see Table 7). These comparatively modest increases might be explained by assuming that the stature of Catalan young people kept increasing after the age of 21. For the Netherlands, Beekink and Kok [23], in fact, observed that, in the first half of the 19th century, the mean physical stature of young males measured at 19 years old and later on at 25 years old increased, on average, by around 5 cm. These scholars also found differences in growth patterns depending on the social class of the recruits and a process of convergence in which young males with the smallest statures at the age of 19 tended to grow more intensely than their peers. Moreover, these authors concluded that, compared to the physical stature of the recruits measured at the age of 25 years old, “the height at age 19 is a more sensitive indicator than adult stature for the circumstances in which a child grew up” [23] (p. 210).

Table 5 and Table 6 show that the relationship between sibship size and height is always negative for cohorts with conscripts measured at 19 years of age. However, the same is not valid with the height of recruits at age 20 and 21. To test for the potential impact of these differences, we have included in our baseline regressions interactions between our variable of primary interest (sibship size) and the recruits measured at the age of 19. The results we obtained from these regressions were not statistically significant. In other words, for the recruits measured at 19 years of age, the relationship between the number of siblings and physical stature is not significantly different from the rest of the sample, suggesting that the age of measurement has little capacity to explain cross-cohort variability.

Another and more important source of heterogeneity in our results might be related to contextual factors. There is enough evidence to show that the sibship size and height relationship varies across both space and time (see Section 1). This evidence directs our attention to the conditional resource dilution hypothesis, which advocates a more flexible approach than the resource dilution model. The flexibility of this new approach lies in the fact that it considers factors such as “economic conditions, cultural norms and practices, and family and gender systems” to understand better how and why sibship size differs within societies [28] (p. 524). It also emphasizes the time dimension as the amount and distribution of parental resources among their children vary from one period to another. Moreover, it points out that parents may not be the only source of resources in a family or household.

All these previous factors help to explain the heterogeneity we found in our results when comparing different birth cohorts. For example, the economic and environmental conditions in which the conscripts’ cohorts were born and grew up differed over time. In the 1860s, Igualada experienced a profound industrial depression and only went consistently back onto its path towards growth in the early 1890s. However, by the late 1880s, the town’s economic and environmental context had improved relative to the early 1870s. As a partial result, the conscripts born in the late 1880s were taller than those born in the 1870s. The available data on mortality go in the same direction: infant mortality rates (1q0) dropped from 173‰ to 101‰ between 1870 and 1890 [77]. Consequently, the amount and the distribution of resources available at the family level are likely to have varied from one group of birth cohorts to another, which, alongside other transformations, might have influenced the association between the sibship size and height throughout the period under consideration. In addition, the family and household composition might also have been affected by changing economic circumstances and other transformations associated with them.

Within the framework of this study, compositional characteristics are, in fact, likely to be the most relevant factors explaining changing patterns in the relationship between sibship size and height across birth cohorts. By compositional characteristics, we mean that the birth cohorts we are considering had a particular composition regarding, among other factors, the mean number of siblings, the percentage of conscripts with literate fathers and the proportion of recruits living in families in which the father was a non-manual worker.

**Figure 8:**
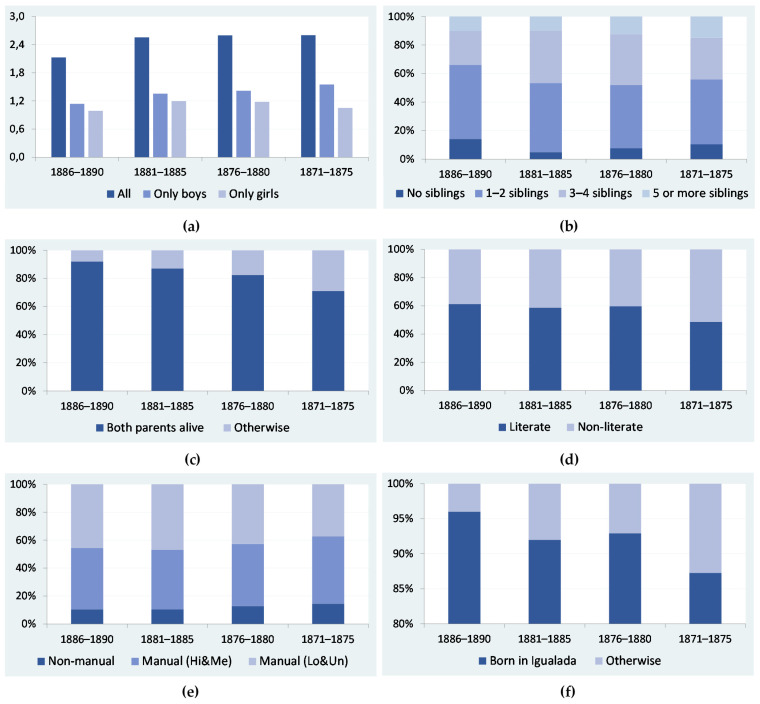
Composition of the birth cohorts by categories. (**a**) Mean number of siblings per recruit. (**b**) Distribution of siblings. (**c**) Parental situation of the recruit. (**d**) Education of the recruit’s father. (**e**) Occupation of the recruit’s father (Hi&Me: high- and medium-skilled; Lo&Un: low-skilled and unskilled. (**f**) Place of birth of the recruit. With the exception of Figure 8a, the information is presented as a % of the total number of observations. Sources: see text in Section 4.

Figure 8 displays information on the composition of the birth cohorts. It shows that, in specific categories, the composition may substantially differ from one cohort to another, with statistically significant differences in general (see Table 8). To give an example, the percentage of conscripts with both parents alive was 92 per cent in the cohorts of conscripts born between 1886 and 1890, but 10 points lower in the cohorts of young males born between 1876 and 1880, and a mere 71 per cent in the 1871–1875 birth cohort (see Figure 8c). Of course, these differences can be explained by contextual factors, as the economic and environmental conditions of Igualada were better in the late 1880s than in the 1870s. Nevertheless, some of these differences might be considered an artefact, arising from the method we have deliberately been using to build up the structure and characteristics of the recruits’ families. As explained in previous sections, this method selects different groups of consecutive cohorts of recruits born between 1871 and 1890, matching them to their families by using a time-invariant census, namely the 1890 local population census. To continue with our example, it might be the case that the percentage of parents alive was lower in the birth cohorts of 1876–1880 and even in the birth cohorts of 1871–1875 simply because they were more distant from the population census of 1890. For these cohorts, we reconstructed the family characteristics of the recruits when they were between 10 and 14 years old and between 15 and 19 years old, respectively. In contrast, for the cohorts of conscripts born between 1886 and 1890, family reconstruction was performed for when they were between 0 and 4 years old. Using this procedure, it is perhaps not surprising that the probability that both parents were alive was higher in the latter than in the former groups of cohorts.

Unfortunately, with the current information at our disposal, it is impossible to disentangle which part of the cross-cohort differences in composition should be attributed to contextual factors and methodological issues. We can conclude the same when considering cross-cohort heterogeneity in the relationship between the number of siblings a recruit had and his biological standard of living. To adequately address this issue, we should compare the family reconstruction we performed using the 1890 local population census with that obtained using previous censuses, starting with the 1870 census to reconstruct the family circumstances of the cohorts born around that year. Regrettably, to the best of our knowledge, this and other subsequent population censuses are not preserved or are incomplete for the period 1870–1885. Thus, at the present stage of research, we have only been able to test for whether, apart from sibship size, other family components influenced the recruits’ height and, based on this information, we strive to infer some of the potential effects that these components may have had on the observed variability across cohorts.

Table 9 is designed as a preliminary approach to this issue. It makes it clear that the outcomes in terms of sibship size and height may significantly differ depending on the parental characteristics of the family in which the focal conscript was born and grew up. For example, according to the data from the local population census of 1890, recruits from families with both parents alive and a literate father working in a high- or medium-skilled manual occupation had more siblings than the rest, with statistically significant differences. The same applies when we look at the mean height of recruits, as well as in the case of occupations. In this latter case, we analyzed the variances (ANOVA), the results of which for the occupational groups show a statistically significant difference between the means of the three occupational groups analyzed for both sibship size and height. We also computed Bonferroni multiple comparisons that use pairwise comparisons, adjusted by multiple comparisons between each group. In this Bonferroni comparison of sibship size by occupation, statistical significance was observed between manual high-/medium-skilled and manual low-skilled/unskilled (*p* = 0.006). With respect to height, we also found statistical significance for non-manual and manual high-/medium-skilled (*p* = 0.002) and non-manual and manual low-skilled/unskilled (*p* = 0.000). In this exercise, we adjusted the *p*-values using the Bonferroni correction.

What about when we consider birth cohorts? Table 10 provides the answer. This table includes compositional characteristics of the recruits’ families. It is derived from Table 5, now presenting information on our independent control variables. As in Table 5, we estimated two different specifications depending on whether sibship size enters the regressions as a continuous variable (panel 1) or as a categorical variable (panel 2).

We interpret the results from Table 10 in the following way. Firstly, cross-cohort heterogeneity remains when we look at their compositional characteristics. This heterogeneity is particularly apparent when we consider the statistical significance of the control variables. While, for most of these variables, no statistical significance is obtained, there are cohorts in which the parental situation of the recruits and the recruits’ father’s occupation reach statistical significance. For example, in the birth cohorts of 1876–1880, conscripts with the two parents alive were almost 3.4 cm taller than those with a single parent or without parents. Perhaps surprisingly, the level of education of the conscripts’ father, as captured by the father’s literacy, did not significantly affect the height of the conscripts in any birth cohort. Other studies focusing on the Mediterranean regions of the Iberian Peninsula between the mid-19th and early 20th centuries obtained an opposite result [25,78].

Secondly, the occupation and skill level of the recruits’ father were consistently explanatory as a determinant of physical stature. Persistently, and for all the groups of cohorts, we observe that low-skilled and unskilled non-manual workers had a physical stature between 2.5 and 3.0 cm lower than manual workers, with a statistically significant difference. This finding requires some comments. In a recent article on two municipalities in the Netherlands in the first half of the 19th century, J. Kok, E. Beekink and D. Bijsterbosch [28] pointed out the importance of the socioeconomic status and the specific conditions of the workers, both for interpreting the influence of family size on height and for weighting the role of rural and urban environments in the stature of young people. According to the authors, “growing up in a town seemingly had a negative effect on height, and this effect remained after controlling for period, food prices, social class, religion and literacy. However, (…) the specific conditions of the workers in the town (…) were of most importance. Many of the unskilled laborers were employed in (…) factories, which included heavy child labor” [28] (p. 107).

Our results seem to point in the same direction and, based on these results, we suggest that part of cross-cohort heterogeneity in testing the relationship between sibship size and height might be related to differences in the socioeconomic composition of the birth cohorts. In addition, cross-cohort differences in the parental survival of the recruits may also have played a role. This latter point is worth noting. If we accept that cross-cohort differences in parental survival are not free from the influence of the approach we used to reconstruct families, we should conclude that methodological issues mediate our final results. Ultimately, from the obtained results, we infer that a combination of contextual and methodological factors explain why differences in socioeconomic composition and family circumstances emerge between birth cohorts.

## 6. Conclusions

This paper analyzes the relationship between sibship size and biological living standards, mainly focusing on methodological issues. In particular, it explores whether this relationship might be affected by the methodology used in matching population censuses and military records and, if so, to what extent. By considering the case of a medium-sized industrial Catalan town, we linked male height data for the birth cohorts of 1871–1890 with time-invariant information obtained from the local population census of 1890. The econometric tests we carried out show that, after controlling for a series of parental variables, the association between the physical stature of a young male and the number of siblings that cohabited with him was never statistically significant. However, these tests also highlight a remarkable heterogeneity across cohorts in other areas of interest. For example, depending on the birth cohort considered, the sign of the relationship between sibship size and height could be positive. At the same time, the birth cohorts in which this relationship was negative also presented substantial heterogeneity regarding the pattern of this association.

An in-depth analysis of the data at hand allows us to conclude that compositional factors mainly explain cross-cohort heterogeneity. Indeed, the reconstruction of the family characteristics of the birth cohorts of 1871–1890 using the 1890 local population census shows that aspects such as the mean number of siblings per recruit, the parental presence in the families or the socioeconomic orientation of recruits’ fathers may substantially vary from one cohort or group of cohorts to another. We attribute some of these differences to contextual factors. However, we hypothesize that the method used to match data could play a role in explaining compositional differences across birth cohorts. While data availability prevents us from disentangling the relative importance of methodological issues on compositional factors, our econometric exercises suggest that the occupation of the recruits’ father and parental survival had a statistically significant influence on the biological living standards of the young males. Differences in parental survival appear to be, in turn, very sensitive to the method used to reconstruct families.

This last statement should not lead to the conclusion that the use of a single population census to construct time-invariant family structures has to be rejected when no other censuses and sources are available. It is not always possible to carry out family reconstitutions through longitudinal methodologies based on continuous information on individuals. While they do not provide information as relevant as active registers, population censuses are a second-best alternative for overcoming the difficulties associated with the availability of sources in historical populations [1,16,31], even when they provide information for a single point in time. Our study suggests that, in these latter cases, prevention and careful analysis of the data at hand are even more necessary than they usually are, simply because the relationship between sibship size and height might be affected by the methodological decisions made in the initial steps of research, such as cohort selection and matching the data.

As well as its methodological contribution, this study adds further evidence to other general issues. In particular, it supports the conditional resource dilution hypothesis by stressing the importance of contextual factors and the role of confounding elements in the relationship between family circumstances and the outcomes of children and young people. The results of this study suggest that socioeconomic factors and parental circumstances might be more critical than sibship size in explaining biological living standards. Of course, and as general warning, our conclusions call for particular caution as they are based on information for a single locality in a particular period and by considering time-invariant family composition for time-variant groups of cohorts.

## Figures and Tables

**Table 1:** A selection of the historical studies testing the relationship between sibship size and male heights (ordered by year of publication).

Study	Technique	Region/Country	Birth Period	Sources (Height Data)	Sources (Family Circumstances)	Observations (No.)	Height (Ages)	Recruit’s Age When Family Composition Is Considered
Hatton & Martin [14] and Hatton [13]	Tim.	16 localities in England and Scotland	*1923–1937*	John Boyd–Orr Survey 1937–1939		2946 *	2–14	2–14
Öberg [16]	Fam.	Southern Sweden	1821–1950	Military records	SEDD	3651	17–25	Birth to 10
Bailey, Hatton & Inwood [12] and Hatton [13]	Tim.	England and Wales	1892–1897	Military records	Pop. cens. of 1901	2236	<18–21 (mean = 20.5)	*4–9*
Tassenaar & Karel [17]	Tim.	Northeastern Netherlands	*1799–1841*	Military records	Civil registries and tax records	413	19	Birth to Death
Beekink & Kok [23]	Fam.	Central Netherlands	1790–1849, 1795–1860	Military records and records of the civic guard	Civil and population registers, censuses and tax registers	2215	19 and 25	Early life
Mazzoni et al. [20]	Fam.	Northwestern coast of Sardinia	1866–1895	Military records	Civil records	1018	20	Birth to 10
Poulain et al. [24]	Fam.	Central-eastern Sardinia	1853–1935	Military records	Parish, civil and population registers	1432	18–20 (adjusted at 20)	*Birth to 20*
Ramon–Muñoz & Ramon–Muñoz [25]	Tim.	Central Catalonia	1845–1850, 1875–1880, 1905–1910	Military records	Pop. cens. of 1860, 1890 and 1920	988	19–21	10–15, adjusted at 10
Roberts & Warren [22]	Tim.	Minnesota	1917–1918	The Children’s Bureau’s 1918 “Weighing and Measuring Test”	Pop. cens. of 1920	8908 *	0–6	*2–8*
Stradford, Van Poppel & Lumey [26]	Tim.	The Netherlands	1944–1947	Military records of 1969		389,287	18	18
Kok, Beekink & Bijsterbosch [28]	Fam.	Western Netherlands	1800–1879	Military records	Censuses, population registers and HSN	1738	19	Early life
Quanjer & Kok [27]	Fam.	8 of 12 Dutch Provinces	1850–1910	Military records	HSN	3003	19–20	Early life
Galofré–Vilà [21]	Tim.	Northeastern Catalonia	1891–1900	Military records	Pop. cens. of 1905, 1910 and 1915	801	21	10–14

Notes: (Fam.) family reconstitution; (Tim.) particular time point; (Pop. cens.) population census; (*) Boys and girls; Sources: see column 1 of this table. Text in italics = own estimation.

**Table 2:** Number of conscripts and families in Igualada in the selected cohorts.

			Number of Conscripts			
Year of Birth	Year of Recruitment	Age in 1890	Total	With Height Data	With Height Data and Found in the 1890 Population Census	No. of Families
1871–1875	1890–1894	15–19	302	296	220 (74%)	161
1876–1880	1895–1899	10–14	404	389	339 (87%)	289
1881–1885	1901–1905	5–9	645	500	324 (65%)	179
1886–1890	1907–1911	0–4	567	468	274 (59%)	145
Total			1918	1653	1157 (70%)	774

Notes: no data for 1873. Sources: see text in this section.

**Table 3:** Descriptive statistics, description and expected sign on height for the variables of interest included in the full sample.

Variables	Variable Description	Min	Max	Mean	SD	Expected Sign on Height
Dependent variable						
Height (cm)	Height in cm of the focal recruit	142.10	190.00	162.67	6.28	
Independent variables (continuous)						
Number of siblings	Number of siblings that the focal recruit has	0	9	2.47	1.63	(−)
Birth order index (BOI)	Recruit’s birth order/((number of children + 1)/2)	0.18	1.78	1.06	0.38	(−)
Birth to birth interval	Mean birth distance between live siblings (in months)	0	300	43.61	29.05	(+)
Independent variables (dichotomous)						
Number of siblings	Number of siblings that the focal recruit has					
0 siblings	Recruit has no siblings	0	1	0.09	0.28	(+)
1–2 siblings (ref.)	Recruit has 1 or 2 siblings	0	1	0.47	0.50	
3–4 siblings	Recruit has 3 or 4 siblings	0	1	0.32	0.47	(−)
≥5 siblings	Recruit has 5 or more siblings	0	1	0.13	0.33	(−)
Two parents alive	Both recruit’s parents were alive	0	1	0.84	0.37	(+)
Father literate	Recruit’s father can read and write	0	1	0.58	0.50	(+)
Non-manual (ref.)	Recruit’s father’s occupation (HISCLASS: 1 to 5)	0	1	0.10	0.30	
Manual high- and medium-skilled	Recruit’s father’s occupation (HISCLASS: 6 to 8)	0	1	0.39	0.49	(−)
Manual low-skilled and unskilled	Recruit’s father’s occupation (HISCLASS: 9 to 12)	0	1	0.38	0.49	(−)
Unknown	Recruit’s father’s occupation	0	1	0.13	0.34	
Born in Igualada	Focal recruit born in Igualada	0	1	0.92	0.27	(+)

Notes: HISCLASS is the acronym for Historical International Social Class Scheme. When the variables are categorical or dichotomous, the expected sign always refers to the reference group. Sources: see text in this section.

**Table 4:** Descriptive statistics of the control variables.

		Height		Sibship Size	
	N	Mean	SD	Mean	SD
Parents					
Both parents alive	969	162.9	6.0	2.5	1.6
One or both parents dead	188	161.6	7.4	2.2	1.7
Father education					
Literate father	667	163.0	6.3	2.6	1.7
Non-literate father	490	162.2	6.2	2.3	1.5
Occupation					
Non-manual	119	165.0	5.8	2.7	1.9
Manual high- and medium-skilled	446	162.9	6.0	2.7	1.7
Manual low-skilled and unskilled	441	162.1	6.1	2.4	1.5
Birth place					
Born in Igualada	1068	162.7	6.3	2.5	1.6
Not born in Igualada	89	162.3	6.3	2.5	1.6
Birth cohort					
1871–1875	220	162.0	6.8	2.6	1.8
1876–1880	339	162.1	6.1	2.6	1.6
1881–1885	324	162.8	6.1	2.6	1.5
1886–1890	274	163.9	6.2	2.1	1.7

Sources: see text in this section.

**Table 5:** The relationship between sibship size and height by groups of five birth cohorts, 1871–1890 (dependent variable: recruit’s non-standardized height, in cm).

	1886–1890	1881–1885	1876–1880	1871–1875 ^(1)^	1871–1890
	(0–4)	(5–9)	(10–14)	(15–19)	(0–19)
Panel 1: Number of siblings enters the regression as a continuous variable					
Number of siblings	0.262	−0.083	−0.355	−0.221	−0.078
	(0.294)	(0.239)	(0.216)	(0.283)	(0.116)
Controls	YES	YES	YES	YES	YES
Constant	162.5 ***	161.6 ***	161.4 ***	160.8 ***	161.1 ***
	(2.761)	(2.045)	(1.942)	(2.471)	(1.287)
Observations	274	324	339	220	1157
R-squared	0.057	0.042	0.055	0.069	0.052
Panel 2: Number of siblings enters the regression as a categorical variable					
0 siblings	−1.429	3.014 *	0.612	2.213	0.728
	(1.405)	(1.824)	(1.557)	(1.871)	(0.797)
1–2 siblings	Ref.	Ref.	Ref.	Ref.	Ref.
3–4 siblings	0.730	0.343	−1.347 *	−0.603	−0.322
	(1.018)	(0.804)	(0.775)	(1.155)	(0.437)
≥5 siblings	0.217	0.915	−0.023	1.285	0.618
	(1.479)	(1.248)	(1.114)	(1.564)	(0.620)
Controls	YES	YES	YES	YES	YES
Constant	163.1 ***	160.6 ***	160.8 ***	158.8 ***	160.7 ***
	(2.855)	(2.077)	(2.051)	(2.493)	(1.324)
Observations	274	324	339	220	1157
R-squared	0.061	0.051	0.060	0.080	0.054

Notes: *** *p* < 0.01, * *p* < 0.1; robust standard errors in parentheses, controls include the following variables: birth order index (BOI), whether the two parents of the focus conscript were alive or otherwise, whether the father of the focus conscript was able to read and write or otherwise, whether the focus conscript was born in Igualada or not, variables connected to the occupation of the conscript’s father and, finally, the conscript’s year of birth. ^(1)^ No information for the birth cohort of 1873. Sources: see text in Section 4.

**Table 6:** The relationship between sibship size and height by groups of two birth cohorts, 1871–1890 (dependent variable: recruit’s non-standardized height, in cm).

	1889–1890	1887–1888	1885–1886	1883–1884	1881–1882	1879–1880	1877–1878	1875–1876	1871–1874 ^(1)^
	(0–1)	(2–3)	(4–5)	(6–7)	(8–9)	(10–11)	(12–13)	(14–15)	(16–19)
Panel 1: Number of siblings enters the regression as a continuous variable									
Number of siblings	−0.479	0.607	0.262	−0.232	−0.037	−0.221	−0.472	−0.227	−0.415
	(0.591)	(0.447)	(0.387)	(0.384)	(0.371)	(0.337)	(0.332)	(0.339)	(0.363)
Controls	YES	YES	YES	YES	YES	YES	YES	YES	YES
Constant	155.6 ***	169.0 ***	160.6 ***	162.9 ***	161.1 ***	156.9 ***	162.0 ***	164.1 ***	162.9 ***
	(5.772)	(3.987)	(3.659)	(3.046)	(2.853)	(3.120)	(2.494)	(2.538)	(3.049)
Observations	112	107	124	137	118	143	150	117	149
R-squared	0.097	0.078	0.052	0.077	0.042	0.098	0.103	0.073	0.107
Panel 2: Number of siblings enters the regression as a categorical variable									
0 siblings	1.638	−1.551	1.749	3.520	2.551	3.187	−1.354	1.392	0.500
	(2.077)	(2.035)	(2.337)	(2.429)	(3.448)	(2.009)	(2.001)	(2.402)	(1.799)
1–2 siblings	Ref.	Ref.	Ref.	Ref.	Ref.	Ref.	Ref.	Ref.	Ref.
3–4 siblings	−1.086	2.145	0.290	−0.229	1.404	0.402	−2.935 **	−2.303 *	−0.550
	(1.773)	(1.531)	(1.374)	(1.280)	(1.216)	(1.152)	(1.145)	(1.354)	(1.425)
≥5 siblings	−4.054	3.127	3.004	0.571	0.175	1.774	−1.304	0.850	−1.075
	(2.574)	(2.213)	(2.056)	(1.965)	(1.836)	(1.681)	(1.585)	(1.638)	(2.052)
Controls	YES	YES	YES	YES	YES	YES	YES	YES	YES
Constant	152.8 ***	170.5 ***	160.3 ***	162.8 ***	160.3 ***	155.1 ***	162.4 ***	163.0 ***	161.8 ***
	(6.224)	(4.128)	(3.814)	(3.004)	(2.843)	(3.237)	(2.482)	(2.373)	(2.968)
Observations	112	107	124	137	118	143	150	117	149
R-squared	0.116	0.097	0.069	0.092	0.057	0.116	0.131	0.111	0.102

Notes: *** *p* < 0.01, ** *p* < 0.05, * *p* < 0.1; robust standard errors in parentheses; control variables as in Table 5. ^(1)^: The last group includes the birth cohorts of 1871, 1872 and 1874. Sources: see text in Section 4.

**Table 7:** The age of conscripts’ inspection, 1871–1890.

Groups of Birth Cohorts	Recruitment Year	Conscripts’ Age Relative to the 1890 Population Census	Age of Inspection and Height of Measurement	Height in cm	
				Mean	Median (50th Percentile)
1886–1890	1907–1911	0–4	21	163.9 (6.220)	163.7
1881–1885	1901–1905	5–9	20	162.8 (6.080)	162.8
1876–1880	1895–1899	10–14	19	162.1 (6.052)	161.9
1871–1875 ^(1)^	1890–1894	15–19	19	162.0 (6.767)	161.0

Notes: ^(1)^ no data for 1873. Standard deviations in brackets. Sources: see text in Section 4.

**Table 8:** Cross-cohort differences by categories, 1871–1890: *p*-values.

	*p*-Value
Mean number of siblings per recruit	
All	0.0009
Boys	0.0024
Girls	0.0463
Distribution of siblings	
No siblings	0.0015
1–2 siblings	0.1082
3–4 siblings	0.0020
5 or more siblings	0.0000
Parental situation of the recruit	
Both parents alive	0.0000
Otherwise	0.0000
Education of the recruit’s father	
Literate father	0.0234
Non-literate father	0.0143
Occupation of the recruit’s father	
Non-Manual	0.9248
Manual, high- and medium-skilled	0.7597
Manual, low-skilled and unskilled	0.0016
Birth Place of the recruit	
Born in Igualada	0.0040
Otherwise	0.0083

Sources: see text in Section 4.

**Table 9:** Mean number of children and mean height by category, 1871–1890.

	(I) Obs.	(II) Sibship Size				(III) Height			
		Mean	SD	*t*-Test	*p*	Mean	SD	*t*-Test	*p*
Parental situation of the recruit				−2.21 **	0.0274			−2.46 **	0.0140
Both parents alive	969	2.5	1.6			162.9	0.5		
Otherwise	188	2.2	1.7			161.6	0.2		
Education of the recruit’s father				−3.16 **	0.0016			−1.99 **	0.0471
Literate father	667	2.6	1.7			163.0	6.3		
Non-literate father	490	2.3	1.5			162.2	6.2		
Occupation of the recruit’s father				5.04 *** ^(a)^	0.0067			10.7 *** ^(a)^	0.0000
Non-Manual	119	2.6	1.9						
Manual, high- and medium-skilled	446	2.7	1.7						
Manual, low-skilled and unskilled	441	2.4	1.5						
Birth Place of the recruit				0.33	0.5671			−0.57	0.5671
Born in Igualada	1068	2.5	1.6			162.7	6.3		
Otherwise	89	2.5	1.6			162.3	6.3		

Notes: *** *p* < 0.01, ** *p* < 0.05, ^(a)^ F-test. Sources: see text in Section 4.

**Table 10:** Height and some of its determinants by groups of five birth cohorts, 1871–1890 (dependent variable: recruit’s non-standardized height, in cm).

	1886–1890	1881–1885	1876–1880	1871–1875 ^(1)^	1871–1890
Variables	(0–4)	(5–9)	(10–14)	(15–19)	(0–19)
Panel 1: Number of siblings enters the regression as a continuous variable					
Sibship Size	0.262	−0.083	−0.355	−0.221	−0.078
	(0.294)	(0.239)	(0.216)	(0.283)	(0.116)
Birth Order Index	1.370	0.180	0.170	−0.478	0.385
	(1.589)	(0.993)	(0.957)	(1.345)	(0.535)
Birth to birth intervals	−0.009	−0.007	0.000	0.008	−0.001
	(0.012)	(0.016)	(0.013)	(0.013)	(0.006)
With one or both parents dead	Ref.	Ref.	Ref.	Ref.	Ref.
Both parents alive	2.865 *	1.523	3.391 ***	1.524	2.273 ***
	(1.632)	(1.214)	(1.256)	(1.449)	(0.666)
Non-literate father	Ref.	Ref.	Ref.	Ref.	Ref.
Literate father	0.079	0.933	0.023	0.660	0.356
	(0.825)	(0.731)	(0.757)	(1.063)	(0.406)
Non-Manual Occupation	Ref.	Ref.	Ref.	Ref.	Ref.
High- and Medium-Skilled	−3.252 ***	−0.729	−1.418	−2.368 *	−1.817 ***
Manual Occupation	(1.245)	(1.100)	(1.097)	(1.355)	(0.585)
Low−Skilled and Unskilled	−2.530 **	−2.361 **	−2.692 **	−3.296 **	−2.630 ***
Manual Occupation	(1.209)	(1.053)	(1.058)	(1.411)	(0.573)
Born outside Igualada	Ref.	Ref.	Ref.	Ref.	Ref.
Born in Igualada	−2.017	0.544	−0.560	2.555 *	0.437
	(1.959)	(1.265)	(1.291)	(1.381)	(0.696)
Birth year controls	YES	YES	YES	YES	YES
Constant	162.5 ***	161.6 ***	161.4 ***	160.8 ***	161.1 ***
	(2.761)	(2.045)	(1.942)	(2.471)	(1.287)
Observations	274	324	339	220	1157
R−squared	0.057	0.042	0.055	0.069	0.052
Panel 2: Number of siblings enters the regression as a categorical variable					
1–2 siblings	Ref.	Ref.	Ref.	Ref.	Ref.
0 siblings	−1.429	3.014 *	0.612	2.213	0.728
	(1.405)	(1.824)	(1.557)	(1.871)	(0.797)
3–4 siblings	0.730	0.343	−1.347 *	−0.603	−0.322
	(1.018)	(0.804)	(0.775)	(1.155)	(0.437)
≥5 siblings	0.217	0.915	−0.022	1.285	0.618
	(1.479)	(1.248)	(1.114)	(1.564)	(0.620)
Birth Order Index	1.412	−0.161	0.119	0.196	0.391
	(1.533)	(1.010)	(0.960)	(1.356)	(0.536)
Birth to birth intervals	−0.016	0.008	0.003	0.019	0.003
	(0.014)	(0.018)	(0.016)	(0.015)	(0.008)
Without one or both parents alive	Ref.	Ref.	Ref.	Ref.	Ref.
Both parents alive	2.674	1.431	3.405 ***	1.672	2.313 ***
	(1.647)	(1.214)	(1.258)	(1.451)	(0.667)
Non-literate father	Ref.	Ref.	Ref.	Ref.	Ref.
Literate father	0.168	1.087	0.024	0.342	0.301
	(0.830)	(0.744)	(0.758)	(1.066)	(0.406)
Non-Manual Occupation	Ref.	Ref.	Ref.	Ref.	Ref.
High- and Medium-Skilled	−3.215 **	−0.668	−1.480	−2.163	−1.822 ***
Manual Occupation	(1.252)	(1.099)	(1.097)	(1.363)	(0.586)
Low-Skilled and Unskilled	−2.548 **	−2.287 **	−2.588 **	−2.887 **	−2.563 ***
Manual Occupation	(1.216)	(1.057)	(1.060)	(1.421)	(0.574)
Born outside Igualada	Ref.	Ref.	Ref.	Ref.	Ref.
Born in Igualada	−1.802	0.548	−0.659	2.529*	0.404
	(1.969)	(1.269)	(1.297)	(1.383)	(0.696)
Birth year controls	YES	YES	YES	YES	YES
Constant	163.1 ***	160.6 ***	160.8 ***	158.8 ***	160.7 ***
	(2.885)	(2.077)	(2.051)	(2.493)	(1.324)
Observations	274	324	339	220	1157
R-squared	0.061	0.051	0.060	0.080	0.054

Notes: *** *p* < 0.01, ** *p* < 0.05, * *p* < 0.1; robust standard errors in parentheses. ^(1)^ No information for the birth cohort of 1873. Sources: see text in Section 4.
